# *npj Primary Care Respiratory Medicine* broadens its focus to include global respiratory health, tobacco control and implementation science

**DOI:** 10.1038/s41533-017-0044-8

**Published:** 2017-06-22

**Authors:** Kamran Siddiqi, Aziz Sheikh

**Affiliations:** 1 Department of Health Sciences, University of York, York, Y10 5DD UK; 2 Usher Institute of Population Health Sciences and Informatics, University of Edinburgh, Edinburgh, UK

*npj Primary Care Respiratory Medicine* (*npjPCRM*) is dedicated to promoting high quality research in respiratory diseases in primary care that has the most significant impact on human health, globally. With the change in leadership following Paul Stephenson’s unparalleled commitment to establishing *npj PCRM* (and its previous incarnations over the past two decades) as the flagship journal it now is, there is the opportunity to build on the journal’s firm foundations and strategically extend its remit. This editorial is an invitation to colleagues to submit research showing how interdisciplinary science can tackle the rising burden of respiratory diseases. In particular, we are inviting authors to submit work focusing on global respiratory health, smoking and tobacco control, and implementation science.

As in most other health conditions, huge disparities exist in the burden of respiratory diseases across the globe. Eighty-three per cent of the 9.3 million deaths worldwide in 2015 due to chronic lung diseases, lower respiratory infections, lung cancer and tuberculosis occurred in low-income and middle-income countries (LMICs).^1^ There are also differences in their distribution and determinants e.g., chronic obstructive pulmonary disease (COPD) is common among men who smoke in Europe and the United States, while in Asia it is predominantly a disease among women who use biomass fuel for cooking.^2^ If knowledge is an instrument to improve health then the well-described 10/90 gap between disease burden and health research depicts a gross neglect of the health of those living in LMICs.^3^ There are no simple solutions to address this gap. To make progress we need to embrace global health’s vision of equity for all people worldwide, which recognises our interdependence to address health issues that transcends national boundaries.^4^ Medical journals, by publishing new knowledge that is relevant and accessible to all its potential benefactors, can play an important role in closing this 10/90 gap. Access to relevant information can help replace the paternalistic and asymmetrical relationships often found in ‘development aid’ with that of interdependence and sharing of resources–a basis for ‘global solidarity’.^5^ In the International Primary Care Respiratory Group, *npjPCRM* has an ideal partner to extend this invitation to the broad community of global health stakeholders and to become an inclusive forum for debate and discussion relevant to lung health. *npjPCRM* will continue to publish work and the opinions of academics and practitioners working in respiratory medicine irrespective of their geographical location. However, we would particularly like to encourage and support those working in LMICs to submit their best work.

Tobacco use has long been recognised as the single most important preventable cause of most respiratory illnesses. Common conditions such as COPD and lung cancer account for a large proportion of deaths attributable to smoking.^2^ Hence, preventing, stopping and protecting from smoking are key to manage respiratory diseases.^6^ Brief advice by general practitioners encouraging all smokers to quit remains one the most cost-effective intervention in healthcare.^7^ In those with COPD, quitting cigarettes is the most effective intervention in stopping disease progression and reducing associated morbidity and mortality.^8^ Even in lung cancer patients, smoking cessation improves survival, treatment efficacy and quality of life.^9^ While practitioners in primary care and respiratory medicine have long been advocating for strong tobacco control policies and provision of smoking cessation services, their scholarly work has not yet been centre stage in the academic forums of respiratory medicine. Recognising this gap, *npjPCRM* is proactively seeking submissions in this area that reflect the diverse and interdisciplinary nature of work that goes on in tobacco control.

While calls to bridge the evidence-into-practice gap in health and healthcare are not new, implementation science^10^ has emerged more recently as an academic discipline in its own right. The study of methods to promote knowledge uptake into policy and practice is now recognised to be just as important as knowledge generation itself. If medical science is to have an impact it is through showing not just its internal validity, but also its relevance to a diverse ‘real world’ context. With initiatives such as the Standards for Reporting Implementation Studies,^11^ there need not be any compromise in the rigour and reporting of implementation research. Implementation science is distinguished by its interdisciplinary nature and explicit use of theories. Whether it is a trial with real word relevance or an evaluation embedded in implementation, a genuine partnership is required between scientists across different disciplines and those responsible for implementing findings. Similarly, implementation science requires an appropriate conceptual framework often rooted within organisational and professional behaviour change theories. In respiratory medicine, implementation science can take many forms,^12^ such as: observational studies of evidence-into-practice gaps, qualitative research to understand barriers/facilitators relevant to routine delivery of care, process evaluations aiming to understand the mechanisms and contexts; assessing outcomes such as intervention reach, sustainability, and adoption; and use of step-wedge randomised controlled trials and natural experiment designs to evaluate interventions. We recognise that implementation science in any disease area would benefit from different strands of evidence coming together. Therefore, *npjPCRM* would welcome submissions in any of the above genres including studies using methods rooted in social sciences.

At *npj PRCM* we remain resolute in our efforts to publish and celebrate the very best science addressing applied respiratory research questions of direct relevance to primary care. Strategically expanding our remit to encourage submission in global health, tobacco control and implementation science will broaden the base from which we tackle key respiratory challenges and to this end we look forward to receiving your contributions.
